# Estimating Bar Graph Averages: Overcoming Within-the-Bar Bias

**DOI:** 10.1177/2041669520987254

**Published:** 2021-01-20

**Authors:** Hyunmin Kang, Jeayeong Ji, Yeji Yun, Kwanghee Han

**Affiliations:** Department of Psychology, 26721 Yonsei University, Seoul, Republic of Korea

**Keywords:** visualization, graph perception, top-down process, bottom-up process, within-the-bar bias

## Abstract

Although most people are not aware of it, bias can occur when interpreting graphs. Within-the-bar bias describes a misinterpretation of the distribution of data underlying bar graphs that indicate an average or where the average estimation point moves inside the bar when the average of several graphs is estimated. This study proposes and tests two methods based on information processing to reduce within-the-bar bias. The first method facilitates bottom-up processing by changing various graph features, such as presenting confidence intervals, placing boundaries around the graph, and showing cumulative bars with different tones. The second method facilitates top-down processing by instructing participants to estimate the mean based on a dot at the end of each bar. Testing of the first method showed that cumulative bars reduced bias, but the other methods did not. The second method was found to reduce bias. Overall, our results demonstrate that the accurate interpretation of bar graphs can be facilitated through the manipulation of specific graph features and instruction.

A recent study found that medical professionals who make decisions about patients’ blood glucose control misinterpreted bar graphs that accurately showed normal blood glucose levels and made improper adjustments due to a phenomenon known as within-the-bar bias (Okan et al., 2018). This type of bias, initially identified by Newman and Scholl (2012), causes people to mistakenly believe that the data underlying a graph are distributed entirely within the bar. However, for bar graphs that represent averages, the top of the bar is actually the exact center of the data distribution.

People interpret data visualizations such as bar graphs through top-down and bottom-up processing (Pinker, 1990). Bottom-up processing, also known as data-driven processing, is a process in which the visual characteristics of data are memorized and processed, mainly affected by salient features or Gestalt principles. Top-down processing, or conceptually driven processing, is driven by concepts, knowledge, and expectations stored in long-term memory (Raschke & Steinbart, 2008). These two processes work in combination. According to Pinker’s (1990) graph comprehension model, a visual description is composed of visual encoding processing from the objects in a visual array. Visual array refers to the visual representation in the initial unprocessed pictorial format, and visual description is a structural description that expresses a graph; the smallest perceptual unit pertaining to aspects of the graph, such as areas and lengths, is processed from the visual array (bottom-up process). In addition, the visual description is affected by the graph schema in existing knowledge, that is, top-down processing. These two processes are “MATCH” and start “message assembly.” Recently, based on this model, Padilla et al. (2018) proposed a new model for decision making regarding visualization materials that involves both Type 1 processing, which is automatic and uses a small amount of working memory, and Type 2 processing, which is deliberate and effortful. In Type 1, decisions are made based on minimal working memory, focusing on bottom-up processing; Type 2 makes more sophisticated decisions through a combination of top-down and bottom-up processing.

Within-the-bar bias is a product of the way people interpret bar graphs, primarily due to a perceptual salient element, that is, the bar. Previous studies have demonstrated two types of within-the-bar bias. The first affects people’s estimation of the data distribution when a single bar with mean values is presented (Figure 1; Newman & Scholl, 2012). The other occurs when people estimate the average point of several bars that are presented simultaneously (Godau et al., 2016). The two types are similar in terms of bias into the bar but have different characteristics. The bias that occurs with a single averaged bar concerns the likelihood of any single value based on the fact that the observer first conceives a virtual distribution. The perceptual elements of the bar cause people to wrongly perceive the distribution of values in the bar; in other words, they misunderstand the data distribution. Pinker’s (1990) theory can be used to infer the cause of bias. Suppose people look at a single bar, such as Figure 1, and they are provided with information that the bar represents an average of multiple values. People make visual descriptions based on *visible* information, such as the length and shade of a bar. However, that bar graph does not give any visible information related to the distribution. At this time, if the conceptual question relating to the distribution (e.g., what is the probability that Point A is a single value) is presented, people cannot respond because their visual descriptions do not have information related to the distribution. However, as humans are also cognitive misers who seek maximum results using as few resources as possible, the prior experience of “visual objects are likely to contain something” would be an inevitable temptation. Therefore, a visual object (in this case, the bar) is poorly selected as a criterion for judging the distribution, and people make a decision that data are more likely to exist within the bar than outside the bar.

**Figure 1.**
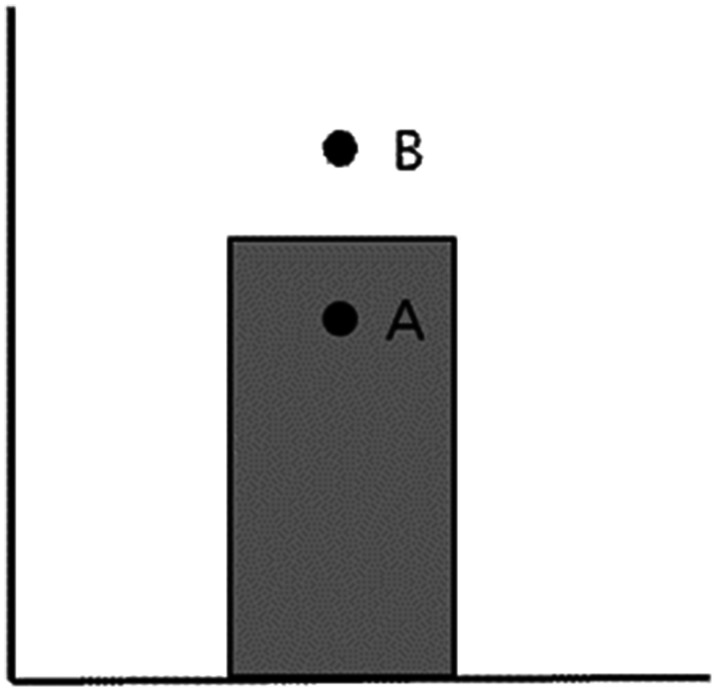
Example of a single bar graph used in Newman & Scholl (2012).

In contrast, estimating the mean of several bars does not require consideration of any hypothetical data distribution. Based on the bars provided, people can determine where the midpoint best represents the edge of the bars. Therefore, when performing the task of estimating the mean of several bars, the visual description formed by combining the information encoded through the visual array with information provided by existing knowledge is sufficient to solve the task. Nevertheless, it seems that the reason for the bias is due to salient features such as the areas, shades, and lines of the bars in the process of comparing and integrating each edge to determine the average point. In particular, if there are many single bars to be compared and the attention needs to constantly shift when determining the average value, there is a high possibility of bias. Pinker (1990) suggested that due to the limitations of short-term memory, the entire visual description is not made at once but is encoded into small subsets. If the encoded subset is not adequate to solve a conceptual question, it could be extracted again by looking at the graph all at once. In the experiment of Godau et al. (2016), bias did not occur when using a point graph but did occur with a bar graph. In the process of comparing multiple stimuli, points on a point graph have the positional information (height) of the object itself. Because of this, it is possible to maintain point information with a small amount of memory. However, in the case of a bar, the information about the top edge of the bar, which is required for the average comparison, is presented along with unnecessary but attention-grabbing information such as the height, shade, and other edges of the entire bar. Visual description groups or separates elements in a graph according to Gestalt principles, such as proximity, similarity, and good continuation (Pinker, 1990). When comparing the edges of multiple bars, it is necessary to remember only the edge, but the other elements of the entire bar are likely to be processed as a criterion for average comparison. A study by Yuan et al. (2019) found that if people want to compare a large set in a bar graph, the visual system does not use the feature that is the most accurate (in a multiple bar task, it is likely to be the edge) but rather the length or area of the bar, which is the less accurate feature. Combining their research with Pinker’s (1990) graph understanding model yields the following. When the average estimation task is presented, people try to determine the average point by comparing edge information in visual description. However, if there are many bars or time pressures (so people respond via Type 1 processing), people will try to use information that is easier to judge (in Yuan et al., 2019, bar length and area), so it is more likely to make an average estimate through other information than edge, and this may be the cause of bias. This study wants to research what information or factors affect people when they estimate an average of bars.

To summarize, both the task of presenting a single bar representing the mean (single bar task) and the task of presenting multiple bars and estimating the mean (multiple bars task) cause within-the-bar bias. However, the reasons for the bias between the two tasks must be considered separately. Thus, attempts to reduce each type should be approached differently.

Previous studies have examined two ways to reduce bias in visualization (Harold et al., 2016; Pentoney & Berger, 2016; Raschke & Steinbart, 2008). The first is to manipulate the characteristics of the graph itself to facilitate bottom-up processing; the second is to support top-down processing by raising people’s awareness of potential bias. These two methods cannot be completely distinguished because graph perception results from the interaction between these two types of processing. However, in this study, facilitation of bottom-up processing is defined as manipulating the elements of a graph to aid in its perception, and facilitation of top-down processing is defined as a method involving experience or memory, not elements of the graph itself.

Most studies have attempted to reduce bias by making changes to graph lines, axes, and shading. Poulton (1985) studied the Poggendorff illusion, a type of bias that affects the perception of a sloping line. If a section of the sloping line is covered by two vertical lines, the slope line is perceived to be more horizontal than it actually is. Poulton suggested that this bias could be greatly reduced by providing a scaled axis. Amer (2005) found that it could also be overcome by drawing grid lines on graphs. In a study aiming to overcome within-the-bar bias occurring with a single averaged bar, Pentoney and Berger (2016) presented a 95% confidence interval on the bar graph and experimented with different shading and line conditions. They found that the shaded with borderline condition showed marginally greater bias than the shaded with no borderline condition and that the bias was almost eliminated if confidence intervals were presented without shading and borderlines. Another study proposed that circle graphs and violin plots are not subject to bias but did not test this with quantitative research (Szafir, 2018).

Top-down processing can potentially be used to reduce bias in graph perception by way of learning or training on graph knowledge. Okan et al. (2012) examined how differences in graph literacy (the ability to understand graphically presented information; Galesic & Garcia-Retamero, 2011) influence the perception of data visualization. They found that those with low graph literacy could not overcome the bias caused by spatial-to-conceptual mapping of the graph, whereas those with high graph literacy could. Raschke and Steinbart (2008) proposed training-based methods, finding that 30 minutes of training on the principles of graph design reduced graph interpretation bias. Such training can also help promote the accurate interpretation of complex visual information. Hegarty et al. (2010) found that training helped people better judge the direction of winds when reading weather maps. However, no study has yet examined whether training can effectively reduce within-the-bar bias.

## Study Purpose

This study examines whether it is possible to reduce within-the-bar bias occurring with multiple bars by facilitating top-down and bottom-up processing. Although some previous studies have attempted to reduce within-the-bar bias occurring with a single bar, few have addressed multiple bars. There are distinct differences between the two types of within-the-bar bias: With a single bar, the interpreter’s task is to understand the underlying distribution, while with multiple bars, the task is to estimate the visible midpoint. Attempts to reduce bias by changing the elements of the graph have obvious limitations in helping people understand the graph’s underlying data distribution when a single bar is presented. Providing direct information about the distribution could be more effective. However, visible perceptual changes can help reduce bias in the case of multiple bars. For example, Xiong et al. (2019) found that in a bar graph with a low average point and no bar-to-bar spacing, the direction of the bias was reversed. One limitation was that the stimulus used in their experiments was perceived as a foreground/background rather than a bar. However, as mentioned earlier, if the elements of the entire bar (height, area, etc.) are the reference points for the average comparison, it is likely that a low bar will cause less bias.

We conducted three experiments to identify the bias in the multiple bar task. In the first experiment, we tried to explore the effect of the length and deviation of the bars on bias. Specifically, a point graph with the same data set was used to confirm whether the bias occurred to a similar extent as the bar graph.

In the second experiment (Experiment 2), we sought to investigate whether factors that could affect the perception of the bar graph influence the bias in the bar. Bias in a multiple bar task seems to occur when only the edges of bars are not remembered as a criterion and other elements of the bar are processed together. Therefore, the degree of bias may be changed through a manipulation that can move attention from the bar or emphasize the edges. In this study, rather than changing the properties (line, shade, color) inside the bar, we added elements to draw attention outside the bar or used auxiliary aids that emphasize the edges that are the basis of judgment. In other words, this is a method of helping bottom-up processing that is encoded as a visual description from a visual array. Specifically, we have identified how presenting (a) confidence intervals, (b) borders, and (c) cumulative graphs affects bias.

Supporting top-down processing will also help people to understand the potential for bias in advance and to suppress their instinct to pull the average in the direction of the axis when estimating the average of multiple bars. This can be facilitated in two ways: explicit methods, involving direct education and training on bias, and implicit methods, which provide indirect training or priming. In Experiment 3, we adopted an explicit method to repeatedly emphasize the edge of the bar and instruct people’s perception of the average.

Throughout the experiments, we tried to extend prior research experimentally. A related study by Godau et al. (2016) presented an anchor line over multiple bars, and subjects judged whether the average was above or below the line. The results demonstrated the existence but not the degree of bias. Accordingly, this study asked people to choose the average location directly. Therefore, we were able to receive participant responses in pixels and compare them between different experimental conditions.

## Experiment 1

We tried to examine what features of the bar graph influence within-the-bar bias. Specifically, a multiple bar task was performed by varying the height and the deviation of the bars, and a point graph task with the same data set was performed to confirm whether the cause of the bias was due to the bar characteristics or equally occurred in the corresponding point graph. In one of Godau et al.’s (2016) experimental conditions, where a deviation of one of the bars is large, a phenomenon has been reported in which bias occurs in the direction of that bar. However, if the deviations within the data are large, the difficulty of estimating the average increases, so it may be a problem related to the limits of absolute judgment rather than the effect of the bar itself. If the bias is due to the difficulty of making an absolute judgment, it will be the same for the point graph as well. However, if the bias does not occur for the point graph, it can be understood that the bias is caused by the characteristics of the bar.

In Experiment 1, we sought to examine the effect of data value characteristics (average height, deviation) on the within-the-bar bias. Point graphs were used as the control condition because they were not affected by within-the-bar bias. A 2 × 3 × 3 mixed design was adopted, with graph type (bar or point graph) as a between-subjects variable and height (low, middle, high) and deviation (small, medium, large) as within-subjects variables. The dependent variable was the degree of within-the-bar bias, measured as the difference between the estimated and actual averages.

Although previous single bar research did not observe any differences in bias according to the orientation of the graph, this study examined whether bias was present for four different orientations. It was not clear if there was a difference in direction for the multiple bar task. This is necessary to confirm whether the occurrence of bias is due to the presence of the bar or the direction, and we wanted to check whether the bias occurred in the vertical and horizontal directions. Therefore, all experimental conditions were carried out with graphs made in four directions, and an additional 2 (graph type: bar, point) × 4 (directions: up, down, left, right) analysis was performed in addition to the aforementioned study design.

### Materials and Methods

#### Participants

The participants included 52 university students (38 women and 14 men) with an average age of 23.20 years (*SD* = 2.55). All subjects were given class credit as compensation for participating in the experiment. Half of the subjects were randomly assigned to the point graph condition and the other half to the bar graph condition. Data from all participants were included in the analysis.

#### Materials

**Figure 2.**
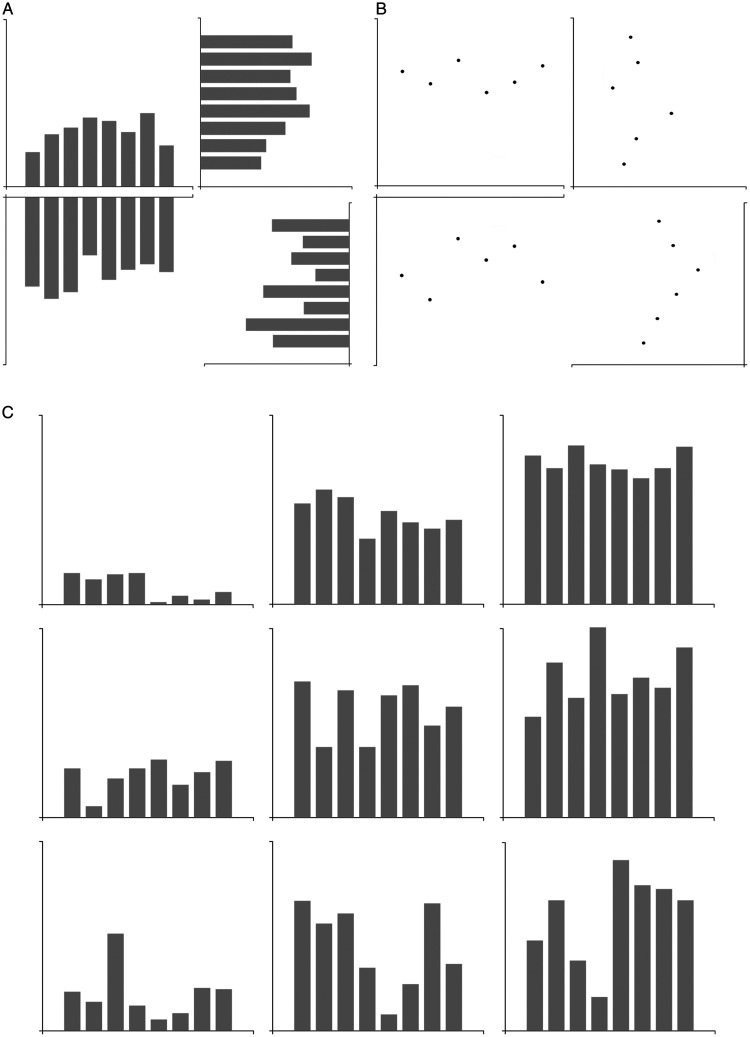
Sample stimuli. (A) Bar graphs, (B) point graphs, and (C) different height and deviation graphs (left column: low average, middle column: middle average, right column: high average, top row: small deviation, middle row: medium deviation, and bottom row: large deviation).

The experimental graphs (Figure 2) were created using the D3 JavaScript library and eight data points randomly generated in Microsoft Excel. Data values were randomly generated according to the height and deviation conditions. The criteria are as follows: Assuming that the entire *y* axis of the graph spanned 15, the average value of the eight graphs was randomly set at 1 to 4 (47–187 pixels; 1.24–4.95 cm) in the low-height condition, 5 to 9 (233–420 pixels; 6.16–11.11 cm) in the middle height condition, and 10 to 14 (467–653 pixels; 12.35–17.28 cm) in the high height condition. For the deviation, random values were made within the range 0.5 to 1.5 (23–70 pixels; 0.60–1.85 cm) in the small condition, 2 to 3 (93–140 pixels; 2.46–3.70 cm) in the medium condition, and 3.5 to 4.5 (163–210 pixels; 4.31–5.56 cm) in the large condition. As can be seen in Figure 2C, nine groups of graphs were produced according to the height and deviation conditions. The graph size was 793 pixels (21 cm) in width and 713 pixels (19 cm) in height. In the graph, there were bars (or points) based on eight data values. The width of each bar was 60 pixels (1.6 cm), and the interval between bars was 20 pixels (0.5 cm). The participants were approximately 60 cm away from the monitor, so the visual angle was 19.9° × 18.0°. All graphs were produced in four directions: up, down, left, and right. A total of 168 graphs were produced with 42 stimuli per direction. In addition, five random graphs were produced separately and shown in practice. The computer-based experimental environment was generated using E-Prime 3 (Psychology Software Tools, Inc., Sharpsburg, PA, USA) on a 21-in. monitor with a resolution of 1,920 × 1,080 pixels.

#### Procedure

**Figure 3.**
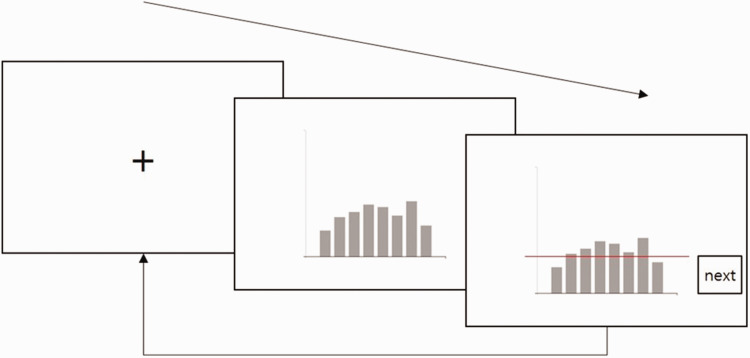
Exercise procedure.

Participants received a verbal explanation of the experiment and filled out a consent form. They were instructed to position their seats in front of the monitor and then to move as little as possible for the duration of the experiment. Participants evaluated five graphs during a practice session to familiarize themselves with the task and the experimental environment. Each graph was evaluated in three stages (Figure 3). First, a blank screen with a fixation point was presented for 1 second to draw the participant’s attention. Second, a bar graph was displayed, and participants were asked to click on the *y* coordinate that they believed to represent the average of all the bars in the graph. Third, a red line was presented across the graph, and participants were asked to adjust their *y* coordinate within 3 seconds to represent the average of all bars in the graph. Participants then pressed the “Next” button to start the next trial. Each experimental block consisted of 42 trials in the same orientation. There were four blocks according to the direction and were presented in random order with a 1-minute break between blocks. The total duration of the experiment for each participant was approximately 25 minutes.

### Results

Data were analyzed using repeated-measures analysis of variance (ANOVA) with IBM SPSS Statistics 25 (IBM, Armonk, NY, USA). Positive values indicated that estimates were biased in the direction toward the *x* axis (within the bar), and negative values indicated that estimates were biased outside the bar. First, the sphericity tests for height (*W* = .25, *p* < .05), deviation (*W* = .84, *p* < .05), and interaction (*W* = .11, *p* < .05) were violated. We thus adjusted the degrees of freedom using the Greenhouse–Geisser epsilon.

As a result, we found that the points and bars showed different biases according to the height and deviation conditions. In the height condition, as the average height increased, the within-the-bar bias in the bar condition was greater than in the point condition. In the deviation condition, as the deviation increased, the bias of the bar and the point increased to the same level.

The three-way interaction between graph type, height, and deviation was significant, *F*(4, 200) = 3.38, $\epsilon_{GG}$ = .46, *p* < .05, $\eta_{p}^{2}\;$= .06. In addition, the two-way interaction between height and graph conditions was significant, *F*(2, 100) = 6.55, $\epsilon_{GG}$ = .57, *p* < .05, $\eta_{p}^{2}\;$= .12, and the two-way interaction between the height and the deviation was also significant, *F*(4, 200) = 20.36, $\epsilon_{GG}$ = .46, *p* < .001, $\eta_{p}^{2}$ = .29. However, the two-way interaction between deviation and graph type was not significant, *F*(2, 100) = 2.19, $\epsilon_{GG}$ = .86, *p* = .13. Examination of the main effects found that the main effects of height, *F*(2, 100) = 64.45, $\epsilon_{GG}$ = .57. *p* < .001, $\eta_{p}^{2}$ = .56, of the deviation, *F*(2, 100) = 17.05, $\epsilon_{GG}$ = .86, *p* < .001, $\eta_{p}^{2}\;$= .25, and of the graph type, *F*(1, 50) = 20.10, *p* < .001, $\eta_{p}^{2}$ = .29, were all significant.

**Figure 4.**
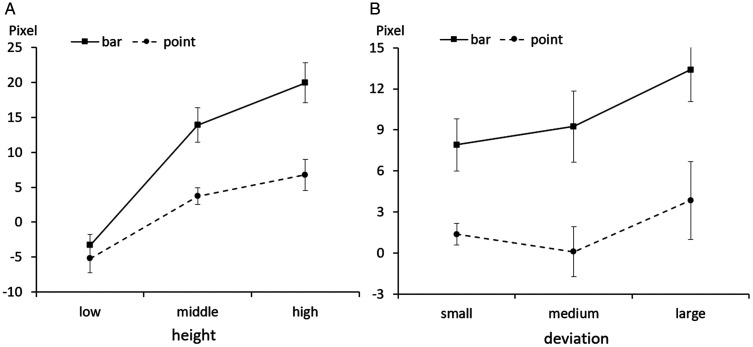
Results of Experiment 1. The actual average is zero; positive values indicate within-the-bar bias. Error bars represent standard errors.

Through the three-way interaction, the bar graph and point graph have different interaction patterns depending on the height and deviation conditions. The two-way interaction result indicated an interaction between the graph type and height, but there was no interaction between the graph type and deviation (Figure 4B). Looking at the interaction between the graph type and height, when the average is low, there seems to be little difference between the point and the bar, but the degree of bias increases significantly for the bar graph as the average height increases (Figure 4A). Specifically, to examine the difference between graph types at each height, a simple main effect test was performed using Bonferroni adjustment. As a result, when the height was low, there was no difference between the bar and the point, *p* = .39, 95% CI [–2.54, 6.36], but when the height was medium, *p* < .001, 95% CI [5.58, 14.76], and high, *p* < .001, 95% CI [6.55, 19.75], the difference between bar and point graphs was significant. In other words, as the height of the bar increased, it could be seen that the degree of the bias in the bar graph increased rapidly, which confirmed that the within-the-bar bias occurred as the size of the visual object increased. It was found that within-the-bar bias was hardly affected by the deviation of the data. As the deviation increases, the difficulty of estimating the average increases, so the bias increases for both points and bars.

**Figure 5.**
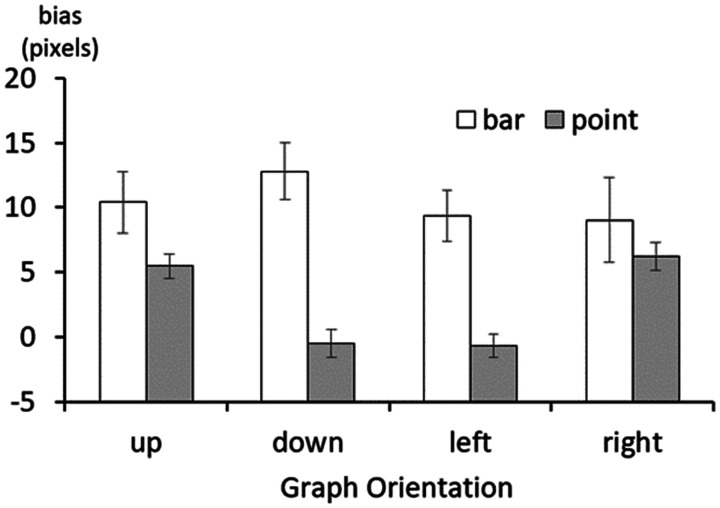
The difference in bias according to the direction and graph type. Error bars represent standard errors.

Next, to examine the difference according to the direction, the graph type (bar vs. point) and graph direction (up vs. down vs. left vs. right) were analyzed using repeated-measures ANOVA (Figure 5). Because the sphericity assumption for the graph direction was violated (*W* = .73, *p* < .05), we used the result of adjusting the degrees of freedom with the Greenhouse–Geisser epsilon. The two-way interaction between graph type and graph direction was significant, *F*(3, 150) = 4.59, $\epsilon_{GG}$ = .86, *p* < .01, $\eta_{p}^{2}$ = .08. The main effect of the condition was significant, *F*(1, 50) = 17.28, *p* < .001, $\eta_{p}^{2}$ = .26, while the main effect of the direction was not significant, *F*(3, 150) = 2.19, $\epsilon_{GG}$ = .86, *p* =. 10. To examine the two-way interaction in detail, we divided the bar and dot graphs to see if there were differences depending on the direction. As a result, no difference in direction was found in the bar graph, *F*(3, 75) = .71, *p* = .55, but a significant difference in direction was found for the point graph, *F*(3, 75) = 15.38, *p* < .001, $\eta_{p}^{2}$ = .38. It can be seen that the graph direction does not affect the average estimation in the bar graph, and in the case of the point graph, the average estimation varies depending on the graph direction.

### Discussion

Summarizing the results of Experiment 1, within-the-bar bias occurred in the bar graph even when the multiple bar task demand actively determined the average point. In addition, the bias was affected by the average height. Given Pinker’s (1990) graph comprehension model, people can process visual objects (in this case, bars) as a whole in perceiving and comparing the edges of the bars to the conceptual question of measuring the average. In this experiment, as the average height increased, the area of the bar also increased. Interpreting the result that the bias increases as the area of the bar increases; it seems that the area of the bar is used as the criterion when performing the average estimation task. We conducted a regression analysis of the actual average of the graph and the degree of people’s bias; it was found to have a statistically significant effect (*β* = .52, *t* = 3.81, *p* < .001).

Our results also showed that the bias increased with the deviation for both the bar graphs and the point graphs. This can be considered the limit of absolute judgment that occurs when estimating the average of large variance data. The bias caused by the deviation should be classified as the bias according to the task difficulty, and not the within-the-bar bias. There is one thing to consider. In Figure 4, when the point graph’s deviation is large, a bias that can be seen as within-the-bar bias occurs. However, this does not appear to be a within-the-bar bias. According to Godau et al. (2016), when there is one bar with a large deviation among 10 bars, the bias occurs in the direction of the bar with a large deviation. Note that in the stimuli used in this study, when the average height is low and the deviation is large, most of the data points are low, but one or two high data points occur. In addition, when the average height is medium or high, most of the data are medium or high, but one or two low data points occur. Therefore, if bias occurs in the direction of the bar with large deviation, bias will occur in the outer direction of the bar under low average height conditions and in the inner direction of the bar if the average height is medium or high. Looking at the descriptive statistics (Table 1), this result can be confirmed, and it can be seen that the pattern is the same for bars and points. Therefore, the point graph result under conditions of large deviation is not due to within-the-bar bias but to characteristics of the data (direction of data with large deviation).

**Table 1.** Descriptive Statistics for Experiment 1.

Height	Low			Middle			High		
Deviation	S	M	L	S	M	L	S	M	L
Bar	–0.41 (4.17)	–7.91 (8.95)	–3.75 (9.44)	11.88 (11.5)	13.22 (13.91)	16.56 (12.34)	10 (12.66)	22.41 (17.18)	27.35 (13.69)
Point	1.81 (5.24)	–5.31 (15.31)	–9.87 (11.85)	2.22 (3.00)	0.41 (7.40)	8.53 (7.79)	2.3 (4.91)	5.15 (5.32)	12.87 (23.86)

*Note.* Values shown are means. Standard deviations are indicated in parentheses.

Moreover, there was no difference depending on the direction of the bar graph. Differences occurred with respect to the point graph, but this study did not examine them in detail because we were interested in the bias that occurs in the bar graph. Therefore, the following experiments were conducted using only the upper and lower graphs, excluding the left and right graphs.

## Experiment 2

Experiment 2 used three types of graphs to examine the effect of manipulation on within-the-bar bias. Each type included a visual element designed to attract attention. The first type was a bar graph with confidence intervals similar to that presented in Pentoney and Berger’s (2016) single bar graph experiment. In a multiple bar task, presenting a confidence interval for each bar can serve to highlight the top edge of the bar representing the data value. In the process of perceiving, remembering, and comparing the bar edges to estimate the mean, the bias can be reduced if the edges can be processed separately owing to the existence of confidence intervals. However, if the entire visual object of the bar is still processed as a whole, the bias is expected to remain. The second type of graph provides a boundary to the graph. Díaz et al. (2018) found that changing the minimum graph elements can affect the perception of bar graph values. In their study, it was suggested that a bar presented in a quantized gradient form or with an axis-like line provided at the top of the graph could allow a bar value to be perceived more accurately. If these manipulations help in perceiving a single value more accurately, the degree of bias in the multiple bar task can also be affected. However, a quantized gradient form is considered unusual in real life, so using a straight line on the top right of the graph to create a boundary is applicable to most graphs, so we tried to use it. However, unlike the estimation of a single data point, the nature of the multiple bars task requires continuous comparison of the bars. Therefore, rather than comparing the average by estimating the position of each value of the bar from the *y* axis, it is more feasible to first roughly compare the edges of the bars and then compare the estimated point with the *y* axis to determine the average. If so, providing the boundary will not be effective. However, if the average estimation task is performed by first accurately recognizing single values and then comparing them, the effect of the border would be expected to appear. The third type of graph is a cumulative-style bar graph (cumulative bar graph) in which the lower portion represented meaningful data. This manipulation was derived from the assumption that estimating the mean of a bar graph is affected most by the bar itself. Cumulative-style bar graphs have one feature: The lower bar and the upper bar share the edge that serves as a criterion for performing an average estimation task. If people try to perceive and remember the edge information to perform the task, they will extract the edge information separately or recognize the upper and lower bars together rather than the lower bar alone. Therefore, it can be expected that the degree of bias will vary depending on the cumulative bar length.

**Figure 6.**
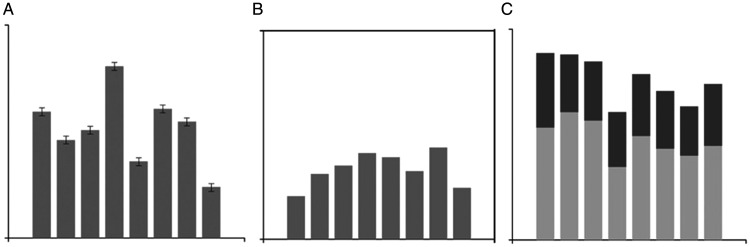
Sample stimuli. (A) Bar graphs with confidence intervals, (B) boundary, and (C) cumulative bar graphs.

In summary, Experiment 2 tested three types of graphs with various features, including confidence intervals, graph boundaries, and cumulative bars with different tones (Figure 6). The results for the bar-only graphs obtained in Experiment 1 were used as the control condition. A 4 × 3 mixed design was adopted with graph type (control, confidence interval, boundary, cumulative) as a between-subjects variable and height (low, middle, high) as a within-subjects variable. The dependent variable was the degree of within-the-bar bias.

### Materials and Methods

#### Participants

The participants were 89 university students (62 women and 27 men) with an average age of 24.72 years (*SD* = 2.80). All subjects were given credit as compensation for participating in the experiment. Participants were randomly assigned to each experimental condition: 29 to the confidence interval condition, 29 to the boundary condition, and 31 to the cumulative bar condition. Eight participants were excluded from analysis: One participant did not complete the experiment because of program error, two had a response rate below 50%, and five were excluded as an outlier. The standard for determining outliers was 2.5 *SD*s in either direction. Thus, 81 participants remained in the analysis, with 25 assigned to confidence interval condition, 25 assigned to boundary condition, and 31 assigned to cumulative bar condition.

#### Materials and Procedure

The experimental graphs (Figure 6) were created using the D3 JavaScript library. All were produced using the same dataset in two orientations. A total of 84 graphs were produced, with 42 per orientation. As for the graph, the height was divided into low, middle, and high in the same manner as in Experiment 1, and in the case of deviation, it was set between 0.5 and 3 without any separate conditions. For graph types, bars with confidence intervals, graphs with boundaries, and bars with cumulative bars were produced. In the case of the confidence interval, a confidence interval of 30 pixels (0.8 cm) was placed around the end of the bar. For the border condition, the *x* axis and *y* axis of the graph were drawn like rectangles to surround the entire graph. For the cumulative bar, different shaded bars were presented at the end of the lower bar, and when the average height was low, the height of the cumulative bar was higher. When the average height of the lower bars was medium, the average length of the lower and cumulative bars was similar, and when the average height of the lower bars was high, the average height of the cumulative bars was low. The procedure was the same as in Experiment 1, and in the case of cumulative bar graph condition, participants were instructed to estimate the average of the lower bars, not the cumulative bars. An additional 30 random graphs were produced separately and used in the practice runs. The experimental environment and procedure were the same as in Experiment 1.

### Results

**Figure 7.**
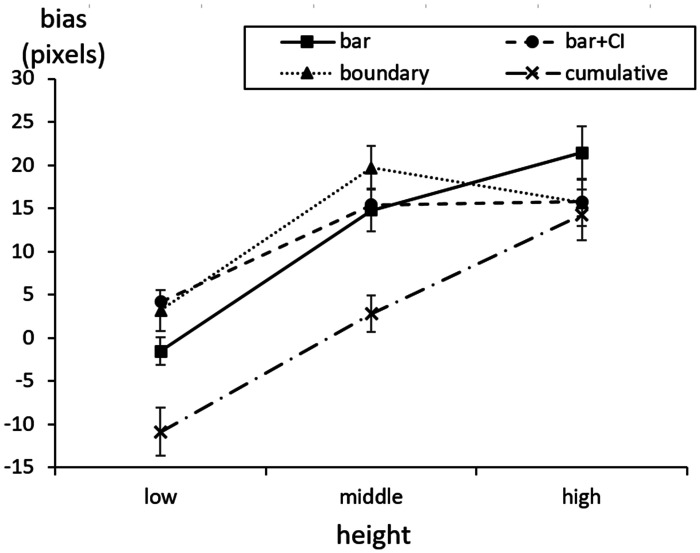
Results of Experiment 2. The actual average is zero; positive values indicate within-the-bar bias. Error bars represent standard errors. CI = confidence interval.

Summarizing the results, it was found that presenting the confidence interval and presenting the border did not reduce the within-the-bar bias, and presenting the cumulative bar decreases the within-the-bar bias (Figure 7). First, we examined whether there was a difference according to the graph directions. Repeated-measures ANOVA on graph type (4: bar, confidence interval, boundary, cumulative) and graph direction (2: up, down) found no interaction according to graph direction and graph type, *F*(3, 103) = 1.62, *p* = .19. In addition, no main effect of direction was found, *F*(1, 103) = 2.23, *p* = .14. Because there was no difference depending on the direction of the graph, we examined whether the degree of bias in the bar depended on the graph type and height.

Because the sphericity assumption for height was violated (*W* = .79, *p* < .05), we used the result of adjusting the degrees of freedom with Greenhouse–Geisser epsilon. The two-way interaction between height and graph type was significant, *F*(6, 206) = 3.01, $\epsilon_{GG}$ = .83, *p* < .05, $\eta_{p}^{2}$ = .08. In addition, the main effect of height, *F*(2, 206) = 150.99, $\epsilon_{GG}$ = .86, *p* < .001, $\eta_{p}^{2}$ = .59, and the main effect of graph type, *F*(3, 103) = 8.90, *p* < .001, $\eta_{p}^{2}$ = .21, were significant. Specifically, the differences between graph types were examined using Bonferroni’s post hoc test. As a result, there was a significant difference between the cumulative bar graph and other types of bar graph (*p* < .05), but there was no difference between other type of graphs. In addition, it was found that the bias in the bar increased as the average height increased (Table 2).

**Table 2.** Descriptive Statistics for Experiment 2.

Height	Low	Middle	High
Bar only	–1.60 (5.20)	14.16 (13.06)	17.55 (15.55)
CI	0.71 (9.00)	14.62 (13.29)	10.21 (11.33)
Boundary	2.64 (7.60)	19.12 (11.01)	18.29 (10.77)
Cumulative	–9.96 (12.22)	4.79 (9.21)	9.27 (9.51)

*Note.* Values shown are means. Standard deviations are indicated in parentheses. CI = confidence interval.

The simple main effect analysis for each point of the average found that the difference between the cumulative bar and other bars was significant when the average height was low (*p* < .05). Even when the height was medium, the difference between the cumulative bar and all other bars was still significant (*p* < .05). However, when the average height was high, the differences between all groups disappeared (Figure 7).

### Discussion

In Experiment 2, we examined the effect on the within-the-bar bias by presenting confidence interval, boundary, and cumulative bar. First, it was found that presenting the confidence interval and boundary did not affect the within-the-bar bias. The confidence interval can make the edges appear, but when comparing many values, it is not possible to obtain only the edge information.

According to Díaz et al. (2018), presenting a line at the end of the graph helps to perceive the exact value of the bar. In the case of boundaries, we hypothesized that the boundary could help in accurately perceiving the value on the *y* axis of the bar. Therefore, if people need the skill to accurately perceive individual bar values when estimating the graph average, the bias is expected to decrease. However, irrespective of finding the exact value, the boundary effect will be ineffective if people approximate the average position of the edges. The experimental results showed that presenting a boundary had no effect on reducing the within-the-bar bias. Therefore, it seems that people make a comparison of the average point roughly through the edge without paying attention to the exact values of the bars.

**Figure 8.**
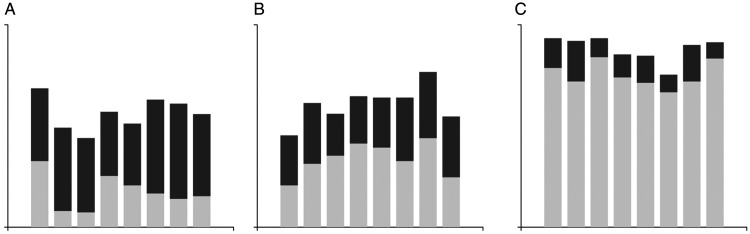
Example of a cumulative bar graph condition. The length of the cumulative bar varies depending on the length of the lower bar.

Last, when the cumulative bar was presented, there was an effect of reducing the within-the-bar bias. One criterion when producing the height of the cumulative bar was that it changed with the average height of the lower bar (Figure 8): When the average height was low, the length of the cumulative bar was relatively long (Figure 8A), and when the average height was medium, the length of the cumulative bar was similar (Figure 8B), while when the average height was high, the cumulative bar was low (Figure 8C). Interestingly, the results showed that when the average was low, bias occurred outside the bar. However, when the average height was high, bias occurred inside the bar (within-the-bar bias). This tells us one thing. When a cumulative bar sharing an edge is presented, the edge information is not extracted separately in the process of comparing the edges of the bar, but the lower bar and cumulative bar are recognized as an entity together with the edge and serve as a reference point for determining the average. Therefore, it appears that bias occurs in the direction in which a longer length and larger area of bar are presented.

## Experiment 3

Experiment 3 tested the effect of instruction to reduce within-the-bar bias. To perform the average estimation task, people need to pay attention to the edges of the bars. Therefore, we tried to determine if instructing to pay attention to the edges can reduce within-the-bar bias. To maximize the effect of the instruction, a cue stimulus (dot) was presented on the edges and participants practiced performing the average estimation task based on the dot. After that, the cue stimulus was removed, and the participants were instructed to perform the average estimation task through edge information as if there was a clue stimulus. It is expected that instructing the estimate of the average through edge information will help to reduce the bias. In this experiment, as in Experiment 2, only the up and down graph directions were used.

A 3 × 3 mixed design was adopted with graph type (control bar, no instruction, instruction) as a between-subjects variable and height (low, middle, high) as a within-subjects variable, as in Experiments 1 and 2. Here, the control bar condition was the bar graph result used in Experiment 1.

### Materials and Methods

#### Participants

The participants were 50 university students (27 women and 23 men) with an average age of 24.12 years (*SD* = 1.92). One participant with a response rate below 50% in the no-instruction condition was excluded from the analysis. Of the remaining participants, 29 were assigned to the instruction condition and 20 were assigned to the no-instruction condition. All subjects were given class credit as compensation for participating in the experiment.

#### Materials and Procedure

**Figure 9.**
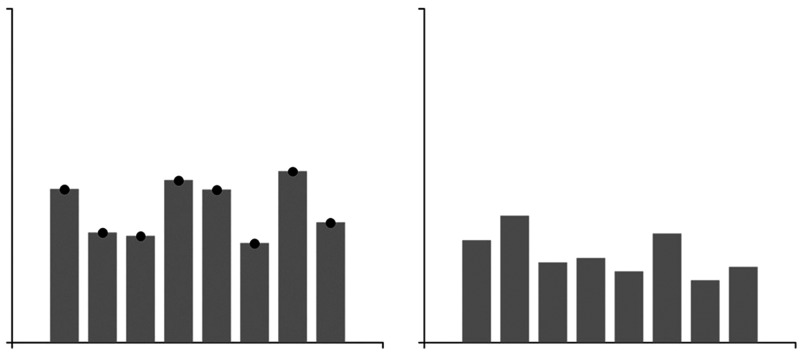
Sample stimuli. (A) Bar graphs with a dot for practice session and (B) bar graphs used for test session.

The experimental graphs (Figure 9) were created using the D3 JavaScript library. Under the instruction and no-instruction conditions, each participant was presented with 40 bar with dot graphs in each orientation (80 graphs), then 20 test graphs in each orientation (40 graphs), for a total of 120 graphs. The experimental environment was the same as in the previous experiments. The dot was located in the middle of the edge and was 19 pixels (0.5 cm) in diameter.

In the procedure of Experiment 3, unlike the previous experiment, a practice session was conducted using a bar with a black dot on the center of the edge. After completing the consent form for the experiment, the participants sat down and adjusted their seats comfortably. Subsequently, the same procedure as in the previous experiment procedure was performed on the screen, but an average estimation task was performed through a bar graph with dots (practice session). The experimenter asked the participants to be as unconscious of the bar as possible and to estimate the average point using only the dots. After completing the practice session, participants took a 5-minute break and then performed the test session. In the test session, we provided different instructions for each condition. Under the no-instruction condition, the experimenter was instructed to estimate the average height of the bars. The experimenter did not give any other instructions and did not explain the dot. If the previous practice had an effect, the bias would be reduced even in this condition.

In the instruction condition, the experimenter instructed the participant as follows: “Unlike the previous practice session, there are no dots on the bar, but think as if there are dots and estimate the average of the bars based on the virtual dots.” At the end of all the experiments, participants were provided postexplanatory instructions. The total duration of the experiment for each participant was approximately 25 minutes.

### Results

The data from the practice session conducted in Experiment 3 were not analyzed. In the case of the control condition, the control condition of Experiment 2 were taken and compared. First, to check the differences by direction, we examined whether there was a difference between the graph type (3: control, no instruction, instruction) and graph direction (2: up, down). The interaction between graph type and graph direction was not significant, *F*(2, 70) = 1.20, *p* = .31, and the main effect of direction was not significant, *F*(1, 70) = .33, *p* = .57.

**Figure 10.**
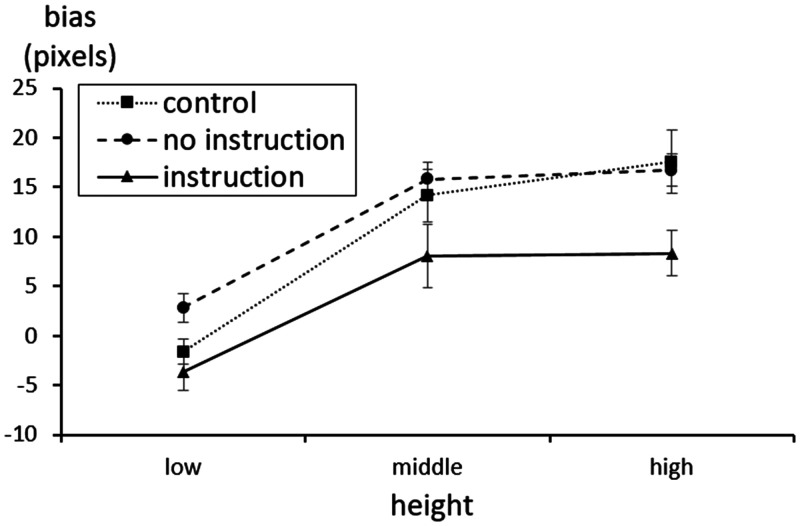
Results of Experiment 3. The actual average is zero; positive values indicate within-the bar bias. Error bars represent standard errors.

In summary, it was found that the within-the-bar bias was reduced in the instruction condition compared with the other conditions (Figure 10). We applied repeated-measures ANOVA to graph type and height. Because the sphericity test for height was not satisfactory (*W* = .75, *p* < .001), the results were used after correcting the degrees of freedom through Greenhouse–Geisser epsilon (Table 3).

**Table 3.** Descriptive Statistics for Experiment 3.

Height	Low	Middle	High
Control	–1.60 (5.20)	14.16 (13.06)	17.55 (15.55)
No instruction	2.83 (8.49)	15.83 (14.39)	16.72 (10.18)
Instruction	–3.68 (6.42)	8.04 (5.48)	8.33 (5.38)

*Note.* Values shown are means. Standard deviations are indicated in parentheses.

Repeated-measures ANOVA analysis showed that the interaction between training and height was not significant, *F*(4, 130) = 1.95, $\epsilon_{GG}$ = .80, *p* = .11, $\eta_{p}^{2}$ = .05. The main effect of graph type was significant, *F*(2, 70) = 5.61, *p* < .01, $\eta_{p}^{2}$= .14: Bonferroni post hoc revealed that the control bar condition (*M* = 10.03, *SD* = 11.27) showed a higher degree of within-the-bar bias than the instruction condition (*M* = 4.23, *SD* = 5.76, 95% CI [–11.39, –0.22], *p* < .05). In addition, the no-instruction condition (*M* = 11.79, *SD* = 14.75) showed a higher degree of within-the-bar bias than the instruction condition (95% CI [–13.56, –1.57], *p* < .01). There was no statistically significant difference between the control bar condition and no-instruction condition. The main effect of height was significant, *F*(2, 140) = 106.25, $\epsilon_{GG}$ = .80, *p* < .001, $\eta_{p}^{2}$ = .60. The low-height condition had significantly lower bias than the middle height condition (*p* < .001) and the high height condition (*p* < .001). There was no significant difference between the middle and high height conditions.

### Discussion

Explicit instruction appeared to have an effect reducing within-the-bar bias. In particular, in the practice session, dots were presented on the bars to serve as reference points. Interestingly, just presenting and practicing bars with dots did not have the effect of reducing bias. Only when the test session included instructions to estimate the average through the edges did an effect appear reducing bias. This tells us that giving instructions that explicitly emphasize the edge rather than an implicit manipulation will reduce the bias.

After the experiment, the participants were asked if they knew the meaning of presenting a black dot. Most participants in the instruction condition (89%) and the no-instruction condition (80%) understood the black dot as pointing to the data to serve as a reference for the task of estimating the average. Therefore, the knowledge information about the mean estimation was sufficiently contained in both conditions, but it seems that the bias cannot be reduced without specific instructions. However, it is necessary to think more about how to give instructions, and in the future, unlike in this experiment, it is necessary to check whether the bias is reduced by giving instructions without practice or dots. Finally, if the height of the bar increased, all conditions showed within-the-bar bias due to the influence of the visual object. In other words, even if people are aware of the black dots and try to make an average estimate consciously, the bar is salient enough to hinder their doing so.

## General Discussion

The purpose of this study was to investigate within-the-bar bias when comprehending a bar graph and making decisions from it. The series of experiments may be summarized as follows: In the first experiment, a point graph and bar graph were compared, showing that the average height influenced within-the-bar bias. The degree of bias changed according to the average value, and it was found that the size of the visual object influenced the bias. In the second experiment, we investigated the effects of the confidence intervals, boundary, and cumulative bar. Presenting confidence intervals that highlight the edge or the boundary and, therefore, may be expected to facilitate accurate perception of the data did not affect the within-the-bar bias. In the third experiment, we provided explicit instruction, and the within-the-bar bias was reduced by using a form that helps top-down processing.

The significance of our study is that the cause of within-the-bar bias in multiple bar tasks was examined based on a model of graph understanding. Within-the-bar bias occurs when the reference point for the average judgment is recognized as the entire bar, not the edge. This can be seen from the cumulative bar graph of Experiment 2. If an object sharing an edge is provided together, the entire object sharing the edge appears to be described as a reference point. This means that when a visual description of a bar graph is generated, objects having continuity are processed as a unit according to the Gestalt principles. Therefore, it can be seen that the bias is close to zero at a middle height, where the lengths of the lower and upper bars are similar from the edge. In addition, when the upper bar is long, bias occurs toward the upper bar, and when the lower bar is long, bias occurs toward the lower bar. Thus, one visual object including both the bars and edge became a criterion for average judgment.

We can also approach the reduction of bias in the cumulative bar with configural shape illusion (CSI; Schloss et al., 2014). CSI is an optical illusion in which a rectangle is perceived as having a relatively longer length if the physical space is adjacent or shared with different visual objects such as an oval or rectangle. In this study, because the lower bar shares space with a cumulative bar, it can be assumed that the length of the lower bar is perceived to be longer than other conditions, and this also reduces the degree of bias and even changes the direction of the bias. However, within-the-bar bias still occurs when the length of the cumulative bar is shorter than that of the lower bar. If the bias decreased consistently across all lengths, this could be explained based on CSI, but it seems reasonable to explain the change in the direction of bias according to the length condition by the ratio of the length of the lower bar to the cumulative bar.

Recently, Ceja et al. (2020) conducted meaningful studies related to the perception of the single bar. They divided the aspect ratio condition of a single bar into three (wide, square, and tall) and presented it to participants for a short period of 0.5 seconds. Participants then undertook the task of reproduction the edge position of the bar. As a result, participants overestimated the edge position in the wide condition (they estimated the edge higher than the actual height) and underestimated in the tall condition. They also reported that this effect occurred in the process of recreating what was remembered because it did not appear if the test bar was constantly viewed and responded. This has implications for the results of our study using multiple bars. In cumulative condition, participants responded an overestimation when the lower bar was short (cumulative bar was long) and underestimation when the lower bar was long (cumulative bar was short). When a horizontal line was presented to estimate the mean, participants moved it near the position where the lower bar met the cumulative bar. At this point, the direction of the bias is determined by the ratio of the lower bar length to the cumulative bar length. If the rate of the lower bar was small, overestimation appeared in the direction of the cumulative bar, and if the ratio of the lower bar was large, underestimation was appeared in the direction of the lower bar. Consequently, in conjunction with the work of Ceja et al. (2020), it can be concluded that in a single bar task, the ratio of the bar itself can affect the length judgment, and when doing a multiple bar task, the ratio of the cumulative and lower bar can affect the average judgment. Of course, it seems necessary to verify in future studies whether this result is limited to the cumulative bar or can be applied to other types of salient objects combined with the lower bar.

There is an irony in the results of Experiment 1. When the height was low, the bias occurred in the direction opposite to the bar (minus value). If the bar is the main cause of bias, it should occur even when a lower bar is present. Because the area of the bar is small, it is possible to explain that it is relatively easy to judge the edge and almost no bias occurs. However, another explanation is needed for bias in the opposite direction. In the study by Xiong et al. (2019), the same result was obtained when using a low bar. However, looking at the descriptive statistics in Table 1, it can be seen that this occurred for a different reason from the study of Xiong et al. (2019). When the height was low and the deviation was low, there was almost no bias and the average estimation point moved very largely outside the bar when the deviation was under medium and large conditions. In the study by Xiong et al. (2019), a graph with a small deviation as well as a small height was used, and in terms of the graphs of this study, it corresponds to a graph with low height and low deviation. To check for statistical differences, a one-sample *t* test was performed to determine whether there was a difference between the zero and the low-height, low-deviation bar, *t*(25) = 1.76, *p* = .09, and point, *t*(25) = –.51*, p* = .62. The results showed no significant difference, and no bias was found. However, when the height is low and the deviation is more than medium, it can be seen that bias occurs in opposite directions to both the bar and the point. This can be understood as not being due to the characteristics of the bar but because of variations in data values. In other words, it can be interpreted as reflecting the difficulty of absolute judgment regarding not only bars but also points.

Despite the value of these results, this study has some limitations. First, several variables were measured only in a binary fashion as to whether they were present, and their individual characteristics did not vary. Cumulative bars were presented in one color only; they could have been presented in multiple colors. Only one type of training was offered, but training could have varied according to variables such as method, duration, and content as well as form (i.e., more explicit training). Each variable that was shown to reduce within-the-bar bias should be independently studied to determine how to maximize its influence. Second, although differences between Newman and Scholl’s (2012) single bar graph and this study’s multiple bar graphs were introduced, they were not directly examined experimentally. In the case of a single bar graph, it would be important to present a hypothetical distribution, and the effectiveness of a manipulation to inform viewers of this distribution should be studied further. Finally, the factors that were shown to reduce within-the-bar bias are difficult to implement in practice, as it is unlikely that anyone would wish to present meaningless cumulative bars in a graph or ask readers to receive training before interpreting their graphs. Given these limitations, future research on more realistic methods to reduce within-the-bar bias is needed.

## Conclusion

Data visualization methods are becoming increasingly prevalent in professional and everyday life, but they are not without drawbacks. Research on memorable data visualization methods (e.g., Borkin et al., 2013) is needed but so is research on graph types that avoid bias and allow for efficient data interpretation. Although many professionals receive training in statistics and graph interpretation, human psychology is still fundamentally subject to biased interpretation. This study demonstrated some methods that can reduce or even eliminate bias, but further research must build on these findings to generate methods for making graphs that can be accurately interpreted. Ultimately, the use of more accurate data representation methods will not only help individuals make better decisions in everyday life but will allow organizations such as businesses and governments to present data more meaningfully and effectively use methods that have a broad social and economic impact.
